# Bevacizumab-Induced Nephropathy Presenting as Crescentic Glomerulopathy

**DOI:** 10.7759/cureus.48787

**Published:** 2023-11-14

**Authors:** Nirmal K Onteddu, Sai Sushrutha Mudupula Vemula, Vivekananda R Areddy, Jayabharath Onteddu, Tejaswi Mabbu

**Affiliations:** 1 Internal Medicine, University of Florida College of Medicine, Jacksonville, USA; 2 Internal Medicine, Gandhi Medical College and Hospital, Secunderabad, IND; 3 Internal Medicine, Sri Venkateswara Medical College, Tirupati, IND; 4 Public Health, University of Florida College of Public Health and Health Professions, Gainesville, USA; 5 Internal Medicine, Sri Venkateswara Institute of Medical Sciences, Sri Padmavathi Medical College for Women, Tirupati, IND

**Keywords:** glomerulonephritis, nephrotic syndrome, thrombotic microangiopathy, nephropathy, bevacizumab

## Abstract

Bevacizumab-induced nephropathy is a common adverse event observed in patients who receive chemotherapy. These patients usually present with hypertension and nephrotic range proteinuria. Thrombotic microangiopathy is the characteristic histologic pattern of bevacizumab-induced nephropathy. However, a few cases reported IgA vasculitis with nephritis as an unusual pattern. In this case report, we describe a patient diagnosed with bevacizumab-induced nephropathy with a distinctive histologic pattern demonstrating focal proliferative crescentic glomerulonephritis with polyclonal immune complex deposition.

## Introduction

Bevacizumab is a humanized monoclonal IgG antibody against vascular endothelial growth factor (VEGF), inhibiting neoangiogenesis. It is used in the treatment of various cancers involving breast, colon, brain, kidney, and lung [1]. Nephrotic range proteinuria (21-64%) and hypertension (3-36%) are the two most common dose-dependent adverse effects of bevacizumab reported in the literature [2,3]. Studies show that a higher dose of bevacizumab (≥10 mg/kg) is associated with a higher risk of proteinuria [4]. Among the patients with nephropathy, thrombotic microangiopathy is the characteristic histologic pattern [3]. A few case reports recently described IgA vasculitis in patients treated with bevacizumab [2,5-7]. Here, we present a case of bevacizumab-induced nephropathy with the histology demonstrating proliferative focal crescentic glomerulonephritis with polyclonal immune complex deposition. To our knowledge, no cases of similar histology have been reported to date, and this is the first case report describing nephrotic range proteinuria with the aforementioned histologic features.

## Case presentation

**Figure 1:**
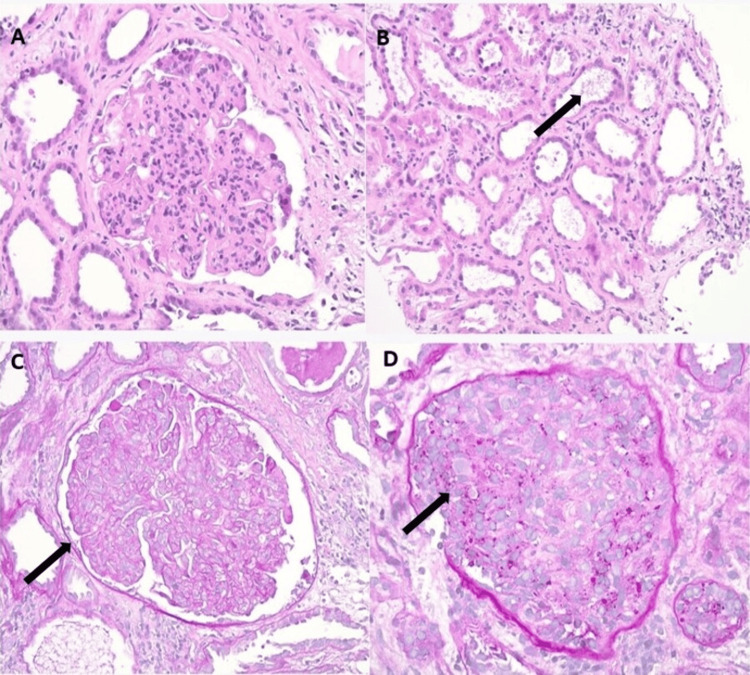
Histopathological imaging (A) Hematoxylin & eosin (H&E) 400x - Endocapillary hypercellularity with infiltrating neutrophils. (B) H&E 200x - Acute tubular injury with cytoplasmic simplification and shredding of cellular fragments into tubular lamina (arrow). (C) Periodic acid-Schiff (PAS) 400x - Global endocapillary hypercellularity with occasional double contours of glomerular basement membrane (arrow). (D) PAS 400x - Glomerulus obliterated by a cellular crescent (arrow) filling Bowman’s space.

A 50-year-old female with a history of colorectal hepatic metastases underwent colonic resection and postoperative chemotherapy with bevacizumab. She presented to the primary care office due to concerns for weight gain, reduced urine output, and poor appetite. Vital signs were within normal limits. Laboratory reports revealed elevated creatine of 6.1 mg/dl compared to her baseline of 1.1 mg/dl. The spot urine protein-creatinine ratio was 28 g mg/dl. Albumin levels were 2.9 g/dl on admission. Renal ultrasound was unremarkable, and the patient was admitted to the hospital for intravenous (IV) fluid resuscitation. In addition, sodium bicarbonate was initiated. Peripheral smear, partial thromboplastin time (PTT), and haptoglobin were unremarkable, excluding hemolysis. Nephrology was consulted and advised to proceed with biopsy, as the patient’s clinical status was not responding to conservative management and had persistent proteinuria. Renal biopsy showed acute immune complex-mediated proliferative glomerulonephritis with a few narrowed glomerular basement membrane duplications accompanied by acute tubular injury (Figure 1). Histopathological staining revealed global sclerosis in three of 14 sampled glomeruli, whereas the remaining demonstrated diffuse endocapillary hypercellularity, including focal neutrophil infiltration occluding the glomerular capillaries. A single glomerulus displayed a cellular crescent. Immunofluorescence staining was positive for 1-2+ IgG, 1+ IgM, 3+ C3, 2+ c1g, and 2+ kappa and lambda. IgA stain was negative. Ultramicroscopic examination showed mesangial and subendothelial electron-dense deposits. Severe effacement of the podocyte foot process was noted. Infectious workup, including HIV and hepatitis B and C, were negative. Rheumatologic workup, including anti-DNA and complement levels, was unremarkable. Ultimately, the renal dysfunction was attributed to bevacizumab, which was later discontinued. During hospitalization, the patient received three daily doses of IV methylprednisolone, followed by oral prednisone 60 mg for a month, and planned for taper pending improvement. In addition, she was started on hemodialysis and scheduled for outpatient dialysis sessions on discharge. After a month, the patient was readmitted for sepsis attributed to a urinary tract infection, and her hospital course was complicated by multiorgan system failure and death.

## Discussion

Bevacizumab-induced nephropathy is secondary to the downregulation of VEGF, disrupting the integrity of glomerular endothelial function and glomerular filtration barrier [8,9]. Physiologically, VEGF-A binds to vascular endothelial growth factor receptor (VEGFR)-1 and VEGFR-2, leading to the production of vasodilatory mediator, nitric oxide (NO), which promotes the endothelium-dependent vasodilation, vascular permeability, adequate renal perfusion, and glomerular membrane integrity [10]. Bevacizumab binds to VEGF-A and blocks the interaction between VEGF-A and VEGF receptors, VEGFR-1 and VEGFR-2 [10]. Disruption in the VEGF signaling leads to podocyte effacement and loss of endothelial fenestrations, ultimately resulting in nephrotic syndrome [10].

Thrombotic microangiopathy is the characteristic pattern of bevacizumab-induced nephropathy, which is manifested as endothelial cell enlargement, podocyte effacement, mesangiolysis, double contoured glomerular membrane, subendothelial edema, and thrombus formation [2,11]. However, in our patient, only a few capillaries demonstrated microangiopathic features. In addition, the pathologic findings leaned more toward the proliferative focal crescentic glomerulonephritis. Other literature-based histologic patterns include IgA vasculitis with nephritis, described in less than 10 case reports [2]. In contrast, our biopsy reports showed polyclonal immune complex deposition, while IgA staining was negative.

Although there are no proper guidelines for the treatment of bevacizumab-induced hypertension and proteinuria, studies have shown that angiotensinogen-converting enzyme inhibitors, angiotensin receptor blockers, and calcium channel blockers are beneficial [12]. As bevacizumab is the most commonly used chemotherapeutic medication, clinicians should be aware of this adverse event for prompt recognition and management. Also, it is a good clinical practice to monitor blood pressure, urine protein, and serum creatinine before and during bevacizumab therapy [13].

## Conclusions

Targeted therapies such as bevacizumab may induce hypertension and proteinuria leading to glomerulopathy. Physicians must be aware of the various possible histologic patterns for prompt disease recognition and management. In addition, periodic surveillance of urinary protein is recommended, and appropriate referral to nephrologists must be made in those patients with proteinuria. Prospective studies are required to further validate the optimal treatment regimen for nephropathy. In addition, an acceptable level of proteinuria can be determined through larger studies while maintaining concomitant chemotherapy.
